# Diffuse cutaneous mastocytosis with novel somatic KIT mutation K509I and association with tuberous sclerosis

**DOI:** 10.1002/ccr3.1607

**Published:** 2018-07-31

**Authors:** Iris M. Otani, Ryan W. Carroll, Phoebe Yager, Daniela Kroshinsky, Sarah Murphy, Jason L. Hornick, Cem Akin, Mariana Castells, Jolan E. Walter

**Affiliations:** ^1^ Division of Pulmonary, Critical Care, Allergy, and Sleep Medicine UCSF Medical Center San Francisco CA USA; ^2^ Division of Pediatric Critical Care Medicine Department of Pediatrics MassGeneral Hospital for Children Boston MA USA; ^3^ Department of Dermatology Massachusetts General Hospital MassGeneral Hospital for Children Boston MA USA; ^4^ Department of Pathology Brigham and Women's Hospital Boston MA USA; ^5^ Mastocytosis Center Brigham and Women's Hospital Boston MA USA; ^6^ Division of Rheumatology, Immunology and Allergy Brigham and Women's Hospital Boston MA USA; ^7^ Pediatric Allergy & Immunology Massachusetts General Hospital Boston MA USA; ^8^ Center for Immunology and Inflammatory Diseases Massachusetts General Hospital Boston MA USA

**Keywords:** blistering rash, diffuse cutaneous mastocytosis, K509I, tryptase, tuberous sclerosis

## Abstract

Diffuse cutaneous mastocytosis (DCM) is a rare but potentially fatal condition when diagnosis and targeted treatments are delayed. This case illustrates the life‐threatening complications in DCM and reviews the currently available treatments. To our knowledge, this is the first report of mastocytosis with somatic K509I mutation and concomitant tuberous sclerosis.

## 1 INTRODUCTION

Diffuse cutaneous mastocytosis (DCM) is a rare and potentially fatal form of cutaneous mastocytosis that is associated with significant mast cell (MC) infiltration of the skin.^1^, ^2^, ^3^ As most cutaneous mastocytosis cases are not associated with significant morbidity or mortality, it is under‐recognized that the DCM type of cutaneous mastocytosis is associated with fatal complications.^1^, ^4^ DCM is characterized by diffuse dense infiltrates of MCs in the dermis, and this high MC burden can result in significant amounts of MC mediator release leading to systemic reactions that can be severe and even fatal.^5^, ^6^, ^7^, ^8^ Given the risk for life‐threatening complications, it is important to consider DCM in pediatric patients with a concerning skin examination (skin with “peau d'orange” appearance, hyper‐pigmentation, positive Darier's sign). A serum tryptase level is an objective screening tool for MC disorders. Early diagnosis can allow physicians and patients to avoid MC degranulation triggers and initiate prophylactic therapy to decrease the risk of MC activation, minimizing complications and improving clinical outcomes.

This case illustrates the clinical course and potentially fatal complications of DCM, and the effective treatment options that are currently available. This particular case is also novel in that it is, to our knowledge, the first reported case of a somatic K509I KIT mutation and the first reported case of concomitant mastocytosis and tuberous sclerosis.

## 2 CASE

A four‐month‐old male infant was referred to an emergency department from his pediatrician's office with fever, irritability, and bullous skin lesions covering a third of the body surface area without mucosal involvement. In the emergency department, he decompensated acutely and developed cardiac arrest requiring cardiopulmonary resuscitation and hemodynamic support with dobutamine, dopamine, milrinone, and norepinephrine. He required mechanical ventilation for acute respiratory distress syndrome and right ventricular heart failure with evidence of pulmonary hypertension, and emergent hemodialysis for acute kidney failure. Due to continued concerns for disseminated infection, he received vancomycin, ceftriaxone, acyclovir, and clindamycin. He developed disseminated intravascular coagulopathy and received multiple transfusions of packed red blood cells, fresh frozen plasma, and cryoprecipitate.

## 3 INITIAL INVESTIGATIONS

While the differential of bullous skin diseases in an infant is broad, key clinical findings that pointed to consideration of mastocytosis were as follows. The infant's skin had a yellowish tinge consistent with the “peau d'orange” skin observed in DCM due to heavy MC infiltration (Figure 2C).^1^ Manipulation of his skin resulted in erythema and hive, indicative of “Darier's Sign” observed in mastocytosis patients.^1^ The patient had been taking increasing doses of famotidine for fussiness attributed to reflux since 2 weeks of age, and 2 weeks prior to his initial presentation, he developed worsening fussiness and nonbloody loose stools. These gastrointestinal symptoms likely reflected activation of mast cells normally present in the gut mucosal lining, rather than benign reflux and diarrhea.

**Figure 1:**
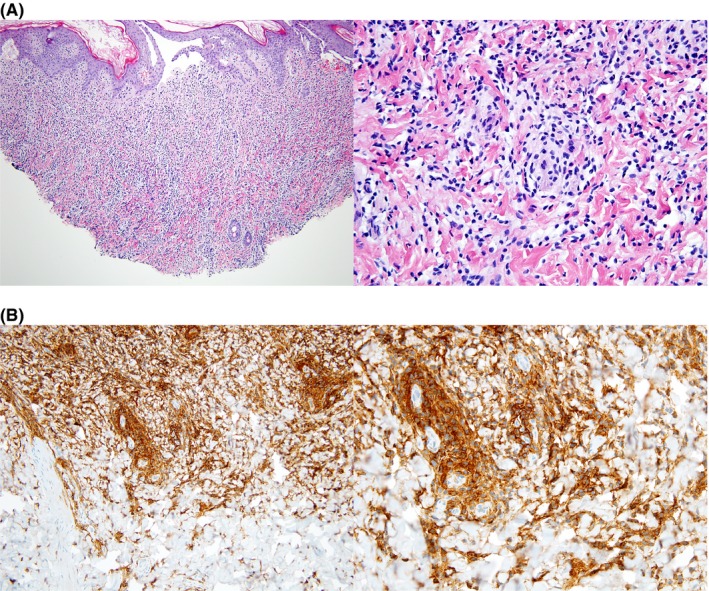
A, Skin biopsy of an abdominal bulla showed a dense dermal infiltrate of mast cells with subepidermal vesiculation consistent with a diagnosis of bullous mastocytosis. B, Immunohistochemistry for KIT (CD117) showed diffuse positive staining of the dermal infiltrate consistent with cutaneous mastocytosis

Skin biopsy of one of the abdominal bullae showed dense dermal infiltrate of MCs with subepidermal vesiculation consistent with a diagnosis of mastocytosis (Figure 1A). Immunohistochemistry for KIT (CD117) showed diffuse positivity of the dermal infiltrate, further supporting the diagnosis (Figure 1B). Direct immunofluorescence was negative for fibrinogen, C3, and immunoglobulins G, M, and A.

## 4 TREATMENT

**Figure 2:**
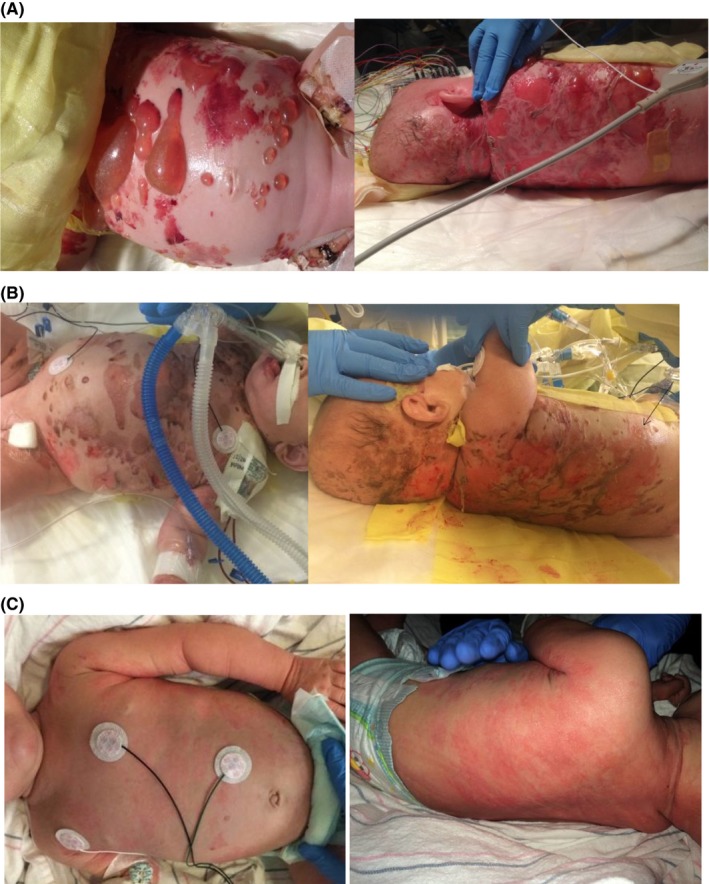
A, Day 1 at our institution. Multiple tense vesicles and bullae filled with serous or hemorrhagic fluid and few large flaccid bullae over the abdomen. Multiple clean‐based large erosions over the back. B, Day 5 at our institution. Chest and abdomen with near complete reattached epidermis, back with healing clean‐based erosions in various stages of re‐epithelialization. C, Day of transfer from our institution back to outside hospital near parents' home (day 31 at our institution). Erythematous patches at sites of healed lesions over the torso

The patient was transferred to our institution for further care. He remained critically ill in the pediatric intensive care unit for a total of 31 days. Initial skin examination showed diffuse blisters and bullae containing serous fluid over the chest, abdomen, back, neck, and posterior scalp (Figure 2A). A total serum tryptase level were 162 nanograms/milliliter (ng/mL), (normal range <11.4 ng/mL). Mature β‐tryptase was <1 ng/mL (normal range <1 ng/mL). Significance of these laboratory results discussed below.

Treatment was initiated with systemic steroids (methylprednisolone 6.3 milligrams (mg) intravenously (IV) every 6 hours), H1‐antihistamine and H2‐antihistamines (diphenhydramine 6.25 mg IV every 6 hours and famotidine 3 mg IV every 24 hours), leukotriene antagonist (montelukast 4 mg per nasogastric tube every 24 hours), proton pump inhibitor (esomeprazole 3 mg IV every 12 hours), and MC stabilizer (cromolyn sodium enteral concentrate 30 mg per nasogastric tube every 6 hours). Precautions were taken to avoid MC degranulation triggers: physical stimuli—friction of skin lesions, heat, cold, pressure, and drugs with MC degranulation potential including vancomycin and radiocontrast media.^9^, ^10^, ^11^

## 5 FURTHER INVESTIGATIONS AND FINAL DIAGNOSIS

Further investigations were performed to investigate the extent of MC involvement. Bone marrow (BM) aspirate smears showed normal MC numbers with normal MC morphology without aberrant CD25+ MCs. BM flow cytometry did not show any evidence of abnormal CD117+/CD25+ MCs. The percentage of CD25+/CD117dim+/CD2dim+/CD3(‐)/CD34(‐) BM MCs detected by flow cytometry was 0.03%. Gastrointestinal (GI) biopsies of the esophagus, stomach antrum, and duodenum did not show increased MC numbers or abnormal MC morphology. The absence of systemic involvement on available BM and GI studies supported a diagnosis of cutaneous mastocytosis, subtype diffuse cutaneous mastocytosis (DCM).

KIT (tyrosine kinase) mutational analysis performed to guide cytoreductive therapy options with tyrosine kinase inhibitors was positive for K509I mutation on skin biopsy and peripheral blood at a level consistent with a somatic mutation (~10%). KIT mutational analysis was performed by extracting DNA from bone marrow aspirate and paraffin‐embedded skin tissue and screened for mutations by Sanger sequencing and high‐resolution melting curve analysis.

Patient has been stable on follow‐up to date 3 years after initial presentation with DCM management. However, as the K509I mutation is also associated with well‐differentiated systemic mastocytosis, if patient develops new symptoms in the future, BM core biopsy may be needed to definitively rule out well‐differentiated systemic mastocytosis.^12^

Further studies were also performed to investigate the subtle clinical seizures that started during hospitalization. Electroencephalography showed evidence of status epilepticus, and brain MRI/MRA showed findings suggestive of hypoperfusion and excitotoxic injury with the additional incidental detection of subependymal nodules and cortical tubers concerning for TS complex. Genetic sequencing revealed a heterozygous c.3397 + 5 G>A splice site mutation in the *TSC2* gene, supporting a diagnosis of TS.

## 6 OUTCOME AND FOLLOW‐UP

**Figure 3:**
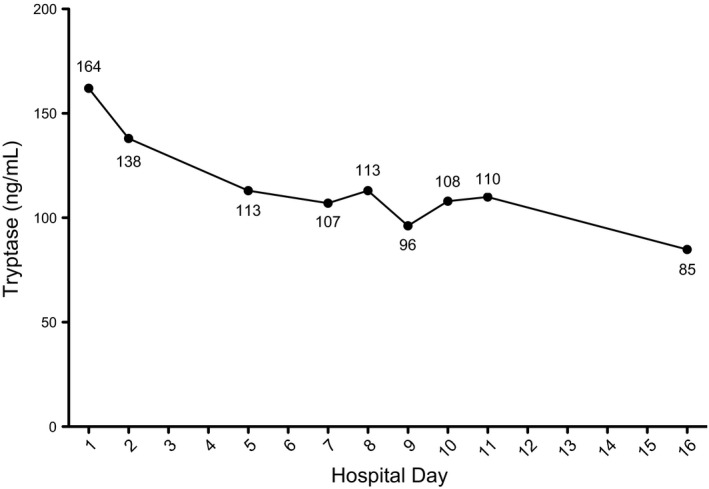
Tryptase levels during hospitalization

With continuation of the above treatment and cautious avoidance of MC triggers, total serum tryptase levels decreased (Figure 3) and skin continued to improve, with re‐epithelialization and only scattered small focal erosions at sites of previous bullae remaining 2 weeks after admission to our institution (Figure 2B). Imatinib was considered but deferred given improvement with the above treatment. At time of retro‐transfer to the referring hospital 1 month after presentation, neurologic examination was notable for an awake, alert infant with development of spasticity in all extremities, and the dermatologic examination was notable for re‐epithelialization and slightly thickened skin with a yellowish hue (Figure 2C).

## 7 DISCUSSION

Diffuse cutaneous mastocytosis is a form of cutaneous mastocytosis that typically presents with cutaneous manifestations at birth or early infancy.^2^, ^13^ DCM arises from a clonal MC expansion and is often due to a gain‐of‐function mutation in *KIT*, which encodes the receptor for stem cell factor (SCF), a potent MC growth factor.^2^, ^13^ Degranulation of cutaneous MCs release MC mediators into the systemic circulation and cause systemic symptoms.^6^, ^14^, ^15^, ^16^

Skin examination is key to differentiate DCM from other benign forms of cutaneous mastocytosis and other bullous dermatological conditions. In DCM, the skin can develop a thickened, leathery appearance with yellowish discoloration, accentuation of hair follicles, and increased folding (“peau d'orange”).^14^ Blistering, flushing, erythema, edema, pruritus, and dermatographism can also be seen.^14^ Darier's sign, elicited by gently stroking the affected skin, is often demonstrated.^14^, ^17^ Darier's sign, although an important screening tool for mastocytosis, should not be elicited in children with cutaneous mastocytosis presenting with extensive cutaneous involvement. This is because skin manipulation in this situation can trigger life‐threatening massive mast cell mediator release associated with extensive cutaneous involvement.^7^, ^10^

A total serum tryptase level is a simple and effective test to screen for mast cell disorders. An elevated total serum tryptase level indicates an aberration in MC function and/or number and should prompt an urgent referral to an allergist.^6^, ^10^ Total tryptase levels can be elevated due to elevations of the inactive protryptase form or the active β‐tryptase form.^18^ Measuring a β‐tryptase level identifies which form is elevated. A normal β‐tryptase level equates to elevation of inactive protryptase and a baseline higher MC burdern.^6^, ^10^ An elevated β‐tryptase level indicates that MCs were recently activated and released β‐tryptase (the half‐life of tryptase is on the order of two to four hours).^18^

Our patient had an elevated total serum tryptase level and a normal β‐tryptase level. Most likely, the β‐tryptase level would have been elevated if it was checked in the two to four hours after presentation (it was checked more than 24 hours after the initial presentation). Acute elevation of beta‐tryptase is a risk factor for developing DIC, which occurred in our patient, as active beta‐tryptase can cleave fibrinogen to fibrin.^19^ The normal β‐tryptase level meant that the total serum tryptase level consisted of predominantly inactive protryptase reflecting an increased risk for severe MC activation.^6^, ^10^

Serum tryptase is also one of the key factors that can help identify children with cutaneous mastocytosis at risk for fatal or near‐fatal mast cell activation events. Previous reports of fatal bullous mastocytosis cases suggest that neonatal onset of cutaneous lesions,^20^ extensive bullous cutaneous involvement,^7^, ^15^ and symptoms of vasodilation and flushing^7^ are common clinical factors associated with fatal mast cell activation events. An analysis of 111 children with cutaneous mastocytosis found that extensive cutaneous disease involving >90% of body surface area and serum tryptase >15.5 μg/L are associated with severe symptoms requiring hospitalization.^10^ The same study found that serum tryptase >30.8 μg/L is associated with severe symptoms requiring management in an ICU.^10^

Early consideration of DCM prompting allergy referral is crucial as early diagnosis allows for avoidance of MC triggers that lead to potentially fatal MC activation. Patients with DCM are particularly prone to MC triggers due to skin manipulation, as perivascular MC infiltrates are present even in areas of skin that appear normal on the surface.^3^, ^16^ Even precipitous temperature changes and routine vaccination can cause severe generalized blistering.^16^, ^21^ Therefore, patients are likely to suffer severe reactions in response to MC triggers ubiquitous in daily life unless they are educated regarding conscious avoidance of MC triggers.

In addition to avoidance of MC triggers, early diagnosis allows for initiation of effective prophylaxis. Prophylactic therapy with long‐acting antihistamines is the mainstay of treatment.^16^, ^22^ Cromolyn sodium, a MC stabilizer, can be administered via an enteral route to treat gastrointestinal symptoms.^23^ Leukotriene antagonists can be efficacious in bullous mastocytosis.^24^ Glucocorticoids can be used in severe cases.^5^, ^25^ Symptom remission has been reported for 2 months to years after PUVA (psoralen, which makes the skin more sensitive to UV light, and ultraviolet A) therapy.^26^, ^27^, ^28^

Furthermore, preceding awareness of a DCM diagnosis can improve treatment in cases where patients experience a life‐threatening flare despite MC trigger avoidance and prophylactic therapies. Epinephrine is the vasopressor of choice when these patients develop cardiovascular collapse, as it can stabilize MCs and prevent further degranulation.^2^ Medications with known MC degranulation potential (including vancomycin and general anesthetics and radiocontrast media) should only be used after careful consideration and discussion with an allergy specialist.^2^, ^16^

This case also has unique genetic features. To the best of our knowledge, our case is the first report of a somatic K509I *KIT* mutation. The majority of *KIT* mutations are somatic,^2^ with D816V (valine for aspartate in codon 816 [exon 17], Asp816Val) being the most commonly seen *KIT* mutation in mastocytosis.^29^ There have been three reports of familial mastocytosis due to germline but not somatic K509I mutation and one report of an individual with a de novo germline mutation.^12^, ^30^, ^31^, ^32^

In addition to the somatic K509I *KIT* mutation, our patient had a mutation in the *TSC2* gene. The TS complex causes benign solid organ tumors and is not known to affect immune cells (including mast cells).^33^ Although our patient had a specific mutation that accounted for the MC clonality causing his DCM, it is interesting to note in the context of our patient's particularly severe and life‐threatening initial presentation that the TSC1 and TSC2 protein‐complex is a tumor suppressor and that mastocytosis (cutaneous and systemic) is a clonal disorder of mast cells.^3^, ^33^ Literature review did not reveal other published reports describing a link between TS and mastocytosis and possible associations between this *KIT* mutation and *TSC2* mutation remain conjecture.

## CONFLICTS OF INTEREST

The authors have no conflicts of interest relevant to this article to disclose.

## AUTHORSHIP

IO and JW: drafted the initial manuscript and revised the manuscript to its final draft. IO: provided Figure 3. RC, SM, and PY: reviewed and revised the manuscript focusing on details of the critical care provided. DK: reviewed and revised the manuscript focusing on details of the dermatological exam and provided Figure 2. CA, JLH, and MC: reviewed and revised the manuscript focusing on details of mastocytosis. JLH: provided Figure 1. All authors approved the final manuscript as submitted.
